# Applications of Dry Film Photoresist in Micromachining: A Review

**DOI:** 10.3390/mi16111258

**Published:** 2025-11-05

**Authors:** Min Zhang, Funa Meng, Xiaoping Li, Wen Zeng

**Affiliations:** 1 School of Emergency Equipment, North China Institute of Science and Technology, Langfang 065201, China; lxp128@yeah.net; 2 Hebei Key Laboratory of Safety Monitoring of Mining Equipment, Langfang 065201, China; 3 School of Mine Safety, North China Institute of Science and Technology, Langfang 065201, China; 15713254748@163.com; 4 Ministry of Education Key Laboratory of Micro and Nano Systems for Aerospace, School of Mechanical Engineering, Northwestern Polytechnical University, Xi’an 710072, China; zengwen@nwpu.edu.cn

**Keywords:** DFR, micromachining, microstructure, mould, mask

## Abstract

Dry film photoresist (DFR) is a solid photosensitive resin film that enables multilayer lamination and rapid patterning at relatively low temperatures. Initially developed for the production of printed circuit boards (PCBs), DFR has demonstrated significant application value in the field of micromachining over the past few decades. This paper systematically introduces the structure and lithography mechanism of DFR, provides a broad classification of its applications in micromachining, and focuses on reviewing the latest progress of different applications, including microstructure creation, mould processing, and sacrificial mask fabrication. Furthermore, this article discusses the current challenges encountered by DFR in micromachining at this point, as well as the key areas that warrant further investigation in future research.

## 1. Introduction

Dry film photoresist (DFR) is a thin film photoresist that undergoes polymerization under ultraviolet (UV) irradiation, enabling rapid patterning. It features uniform thickness and supports adhesion to various substrates and multilayer lamination at lower temperatures, facilitating the creation of embedded structures without compromising material integrity [1,2,3]. Additionally, DFR exhibits excellent biocompatibility and chemical resistance [4,5]. In comparison to the microfabrication based on liquid photoresists (e.g., SU-8), the applications of DFR eliminate the need for expensive equipment and a clean laboratory environment, as well as spin-coating and soft-baking steps, thereby reducing cost and processing time [6,7].

Since the first demonstration of DFR for printed circuit board (PCB) fabrication in the 1970s by DuPont [8], this material has attracted more attention. Especially with the development of microelectromechanical systems (MEMSs), it has found extensive applications in micromachining, such as mould fabrication, pattern transfer, system packaging, and biosensing [1,7,9].

So far, several review articles have been published on the applications of DFR in specific industries, such as optical bonding [10] and electrode manufacturing [11]. However, there are few comprehensive reviews on the lithography mechanisms of DFR and its extensive applications across the micromachining field. In this paper, we systematically introduce the structure and lithography mechanism of DFR, and provide a broad classification and detailed review of its latest application progress in the field of micromachining. Although DFR has been rapidly developed and applied, some problems still remain. We have discussed the key technical challenges faced and research priorities that need to be focused on in the future. We believe that this review article will provide a comprehensive reference platform for researchers and potential industrial application engineers alike.

## 2. Structure and Lithography Mechanism of DFR

### 2.1. Structure and Types

DFR mainly consists of three layers; the middle layer is photoresist and the two outer layers are polyethylene (PE) film and polyethylene terephtalate (PET) film, respectively [12]. Specifically, the PE film serves as a protective layer that isolates oxygen, prevents delamination, and protects against mechanical scratches. Before application, it must be removed to expose the photoresist layer for UV exposure. The PET film functions as a substrate for the photoresist, offering structural support and withstanding mechanical stresses during processing such as coating, exposure, and development to ensure the integrity of DFR. The photoresist, as the core component, shows high sensitivity to specific wavelengths of UV light. Upon UV exposure, certain chemical reactions occur, resulting in a change in its solubility within the developer solution.

DFR can be classified into positive and negative types based on their distinct properties. The summary of comparisons between the two types of DFR is provided in Table 1. Despite its slightly lower lithographic precision compared to positive DFR [13], negative DFR remains highly favoured in the field of microfabrication due to its lower cost, better corrosion resistance, stronger adhesion, and greater flexibility in pattern design [14,15]. These advantages give it greater competitiveness in large-scale processing and cost sensitive projects, making it an important material for preparing complex microstructures. Therefore, this paper focuses on reviewing the advancements in the application of negative DFR.

### 2.2. Lithography Mechanism

The lithography process of DFR primarily involves exposure, development, and film stripping. Focusing on the widely applicable negative DFR [15], under the irradiation of UV light (wavelength range of 300~405 nm), the photoinitiators within the photoresist release free radicals (I*) (Equation (1)). These active radicals (I*) subsequently initiate photopolymerization reactions with the photopolymeric monomers present in DFR (Equation (2)). Through a series of continuous polymeric cross-linking reactions, these radicals (I*) establish stable ester bonds with “-COOH” groups, converting the initially soluble structures into an insoluble, complex cross-linked network that is resistant to the developer solution (Equation (3)). During the following development process, the UV-exposed regions show stable chemical properties and do not react with the developer solution, thus remaining on the substrate. In contrast, the unexposed regions, which contain “-COOH” groups, undergo acid–base neutralization reactions with CO_3_^2−^ ions present in the developer solution and eventually dissolve in it (Equation (4)). The stripping of DFR involves saponification reactions between the UV-exposed DFR and the OH- ions present in stripping solution (Equation (5)). This reaction can effectively break the cross-linked network formed during exposure, facilitating the smooth removal of DFR from substrates.

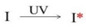
(1)

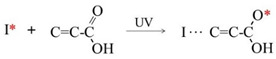
(2)

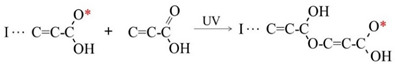
(3)

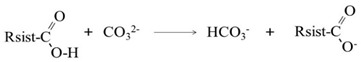
(4)

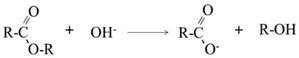
(5)

### 2.3. Characteristics of Micromachining Based on DFR

**Figure 1:**
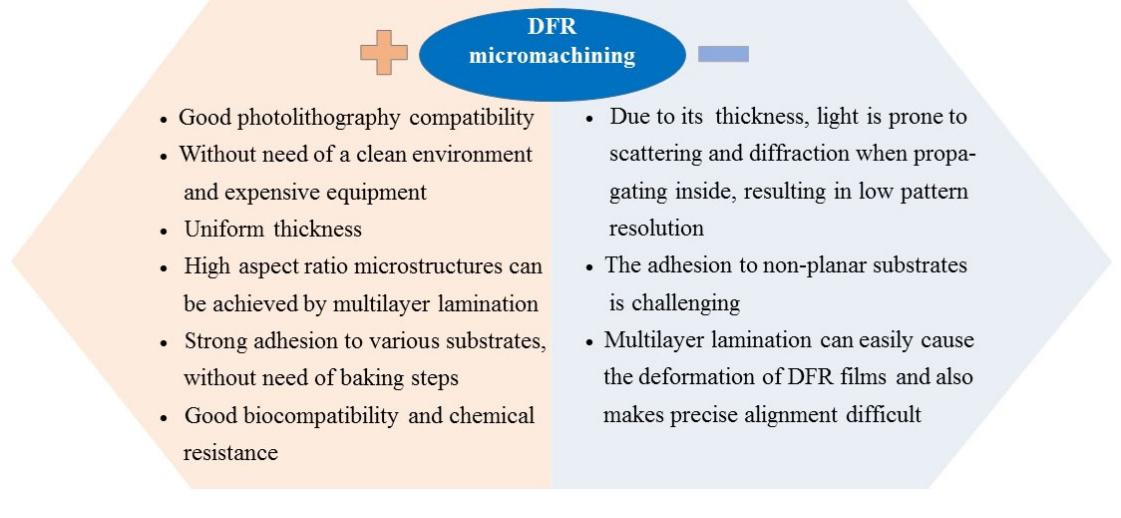
Characteristics of micromachining based on DFR.

DFR-based micromachining has unique characteristics compared with traditional microfabrication processes. Here, we summarize the advantages and limitations of DFR processing as shown in Figure 1. Due to its significant advantages, such as good photolithography compatibility, with no need for a clean environment and expensive equipment, and convenient realization of high aspect ratio microstructures through multilayer lamination, it has been widely applied in the field of micromachining. However, there are also some process limitations, such as low pattern resolution, film deformation caused by lamination, and low precision alignment. Therefore, we will comprehensively review the typical applications of DFR in micromachining in recent years, and discuss the key technological progress made and the challenges that remain.

## 3. Broad Classification of DFR Applications in Micromachining

**Figure 2:**
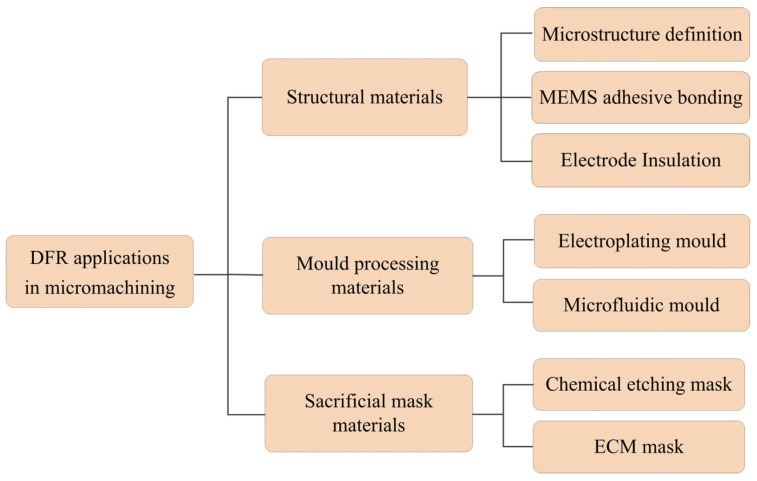
Broad classification of DFR applications in micromachining.

Intense research has led to diverse applications of DFR in micromachining, from microfluidic chips, flexible electrodes to solar cells, exploiting its unique performances. This paper comprehensively summarizes these applications and provides a broad classification, as shown in Figure 2, which mainly includes three categories: structural materials, mould processing materials, and sacrificial mask materials. The following sections will conduct a detailed review and discussion of each type of application.

## 4. Applications of DFR as Structural Materials

### 4.1. Microstructure Definition

This section mainly reviews the typical applications of DFR in the definition of microstructures, including microfluidic chips (including both simple chips and complex 3D microfluidic networks), microcantilevers, and waveguide components.

#### 4.1.1. Microfluidic Chip Definition

Microfluidic chips are capable of conducting reagent analysis with minimal sample volumes, which greatly reduces detection cost and improves detection efficiency [16]. Over the past few decades, they have been widely applied in fields such as biology, chemistry, and medicine. Traditional chip manufacturing employs Si-based chemical etching [17] or SU-8 lithography [18], both of which rely on costly equipment and involve complex procedures.

**Figure 3:**
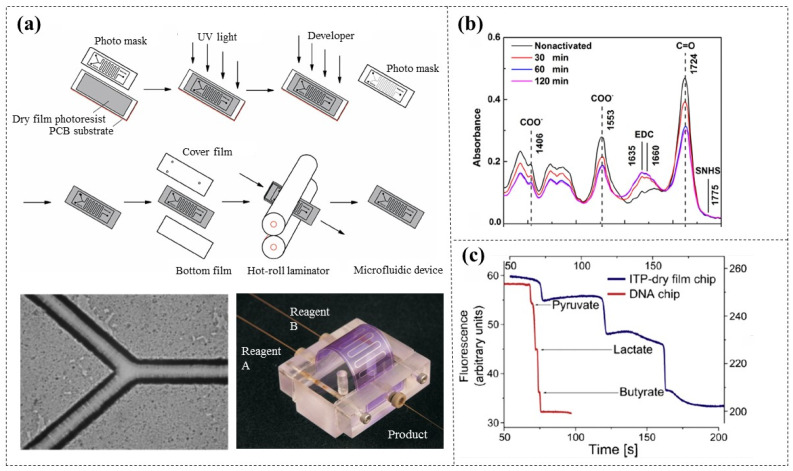
(**a**) A disposable microreactor with simple microchannels [5]. (**b**) Section of IR spectra of DFR before and after treatment with EDC/SNHS activation solution [19]. (**c**) The natural fluorescence effect of DFR chips [20].

Tsai et al. [5] first proposed using photolithography to create simple microchannel patterns on DFR (AF-5075, 75 µm thick) surfaces, which are then sandwiched between two PET films and thermally pressed to fabricate plastic disposable microreactors. To prevent liquid leakage, PDMS sheets were used to provide reliable liquid connections (Figure 3a). This chip processing method is low cost, fast prototyped, and does not require sacrificial materials. Horak et al. [19] demonstrated that the surfaces of DFR (Vacrel 8100, 38 µm thick) can be modified via EDC/SNHS activation to form amine-reactive esters, which can be used for the immobilization of biomolecules. IR spectra tests confirmed that the optimal activation time is 60 min (Figure 3b). This advancement simplifies the detection of biomarkers in clinical samples. Smejkal et al. [20] compared the DFR-based (Ordyl 330, 30 µm thick) microfluidic chips with commercially available glass-etched DNA chips and found that DFR exhibits a natural fluorescence effect (Figure 3c). Fortunately, this fluorescence is relatively low, being compatible with most microfluidic applications using fluorescence detection, which was also confirmed in other scientific literature [21]. In addition, the microchannels defined by DFR have been verified to have good biocompatibility with various living cells and biological reagents [22,23].

**Figure 4:**
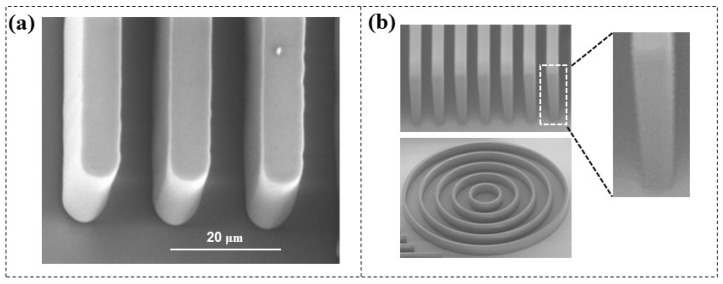
(**a**). SEM images of free-standing bars with an aspect ratio of 4.28:1 [29]. (**b**) SEM images of free-standing bars with an aspect ratio of 7:1 and channel structures with an aspect ratio of 5:1 [30]. The width of the bars is 7 µm, and that of the microchannels is 10 µm.

Increasing the aspect ratio of microchannels can enhance the throughput of reagents within microfluidic chips and improve their flow characteristics. The most common approach involves stacking multiple PDMS [24,25,26] or SU-8 layers [27,28], which requires repeated baking processes. Researchers try to increase the aspect ratio of microstructures through multilayer DFR lamination. Stöhr et al. [29] created free-standing DFR (TMMF, 30 and 55 µm thick) bars with an aspect ratio of 4.25:1 (Figure 4a), while Courson et al. [30] further advanced this work by developing free-standing bars (DF-1050, 50 µm thick) with an aspect ratio of 7:1 and channel structures with an aspect ratio of 5:1 (Figure 4b), although the sidewall profile exhibited a slight negative angle (≈92°). Furthermore, they all concluded that when the aspect ratio is greater than four, the minimum achievable width for a free-standing structure is 7 µm, while for a channel structure it is 10 µm; otherwise, structural collapse may occur. Microstructures with higher aspect ratios and smaller dimensions still face challenges.

**Figure 5:**
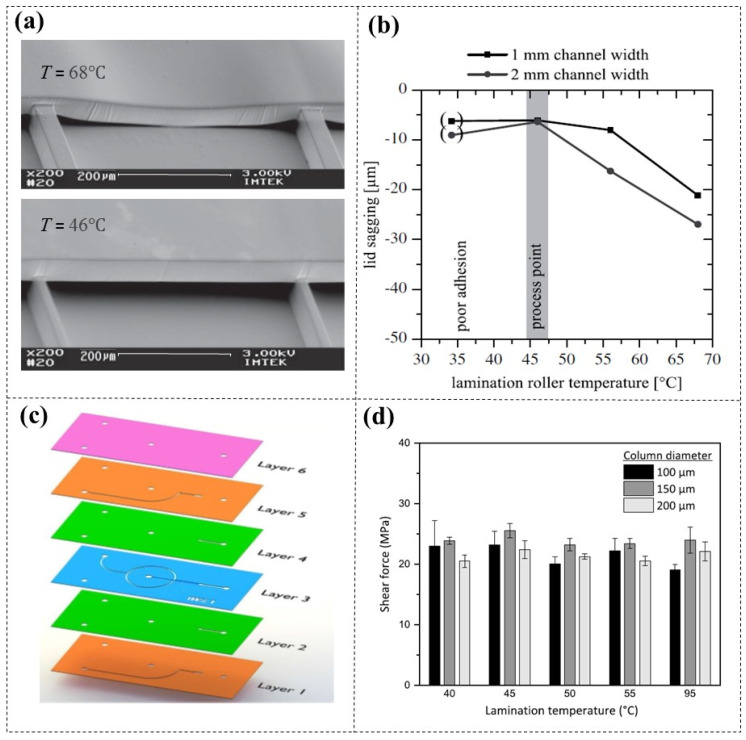
(**a**) SEM images of microchannels at different lamination temperatures, and (**b**) the influence of lamination temperature on sagging of microchannels [35]. (**c**) Schematic diagram of a 3D hydrodynamic flow-focusing device composed of six layers of different patterned DFR, and (**d**) the effect of decreasing layer-by-layer lamination temperature (95–40 °C) on the adhesion of the DFR/DFR interface [38].

Multilayer laminating of DFR with different patterns has promoted the development of highly functional microfluidic networks [31,32]. During the lamination, temperature is a crucial technical parameter. Increasing the lamination temperatures can improve layer adhesion [33,34], but excessive heat may cause DFR sagging and clog lower microchannels [35,36] (Figure 5a). Wangler et al. [35] conducted an investigation into the optimal lamination temperature for preventing DFR (TMMF, 45 µm thick) channel collapse at various channel widths (Figure 5b), and found that adequate results can be achieved between 40 and 50 °C. This conclusion was also confirmed by other researchers [37,38]. El Hasni et al. [38] explored a 3D hydrodynamic flow-focusing device, which was composed of six layers of DFR (SUEX K25, 25 µm thick) with different patterns (Figure 5c). They further analyzed the impact of lamination temperature on the adhesion properties of the DFR/DFR interface. By gradually decreasing lamination temperature from the second layer to the sixth layer (95–40 °C), they found that the shear force between the interfaces changed little, approximately by 22 MPa (Figure 5d). These findings provide the experimental foundation for achieving low-temperature lamination of multilayer DFR.

**Figure 6:**
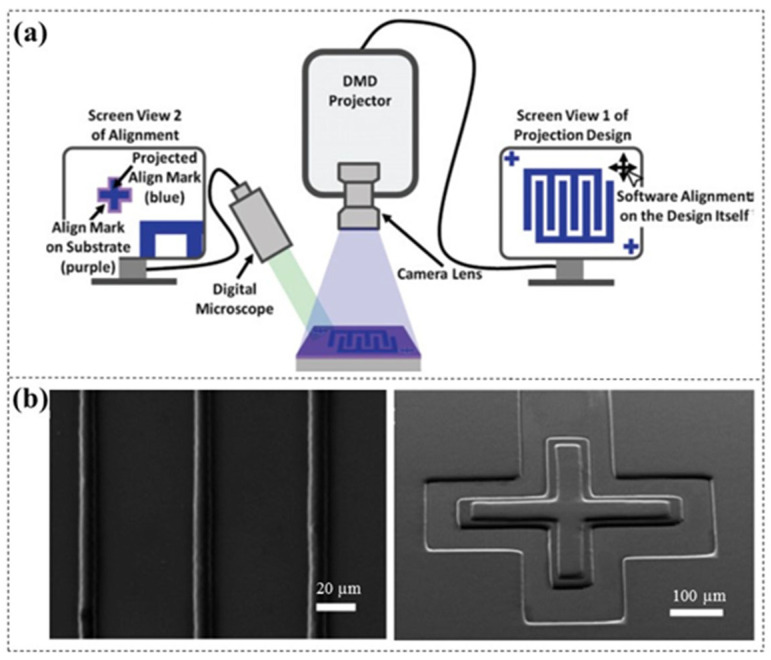
(**a**) Schematic diagram of the direct projection lithography system with software alignment capability and (**b**) SEM images of lines with the minimum width of 10 µm and two-layer crossbar structures with less than 10 µm alignment precision [39].

During the multilayer lamination, precise alignment of patterns between each layer is challenging. Zhao et al. [39] developed a direct projection lithography (DPL) system with software alignment capability (Figure 6a), offering a more precise method compared to the conventional manual [17,18,27] and mechanical alignment techniques [23,31,32,33,40]. By using dual computer displays and software position adjustment, this approach effectively reduces the misalignment induced by manual or mechanical movement, achieving precise alignment of less than 10 µm (Figure 6b). It is worth noting that the alignment accuracy is limited by the projected pixel size.

For microfluidic chip fabrication, DFR can not only be directly used to define microstructures but also for the processing of microfluidic moulds, which will be discussed in detail later.

#### 4.1.2. Microcantilever Fabrication

Microcantilever-based sensors have experienced rapid development following the invention of the atomic force microscopy (AFM) [41]. Researchers developed polymer microcantilevers made from SU-8 photoresist, which have shown high deflection sensitivity and good compatibility with conventional lithography [42,43]. However, issues such as non-uniform spin-coating and plastic deformation of the microscope probe continue to pose challenges for researchers.

**Figure 7:**
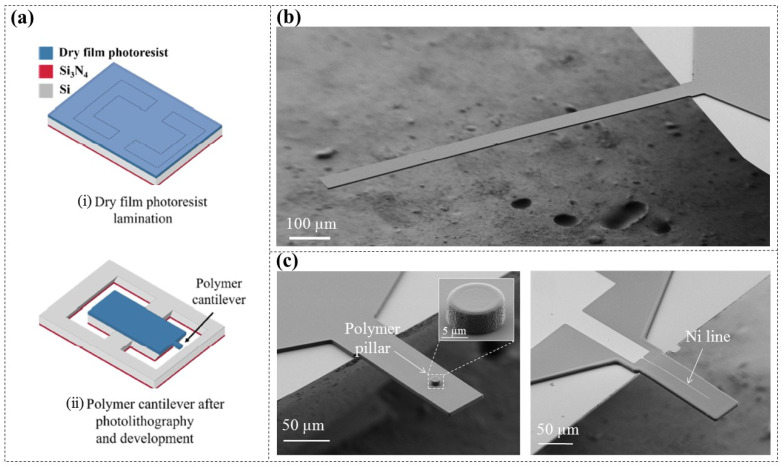
DFR microcantilevers [44]. (**a**) Processing schematic diagram. (**b**) Image of a simple DFR microcantilever that is 1000 μm long and 50 μm wide. (**c**) Images of DFR microcantilevers integrated with a 3D pillar structure (left) and a 1.2 μm wide Ni line (right).

Nilsen’s team initially fabricated DFR-based (ADEX, 5 µm thick) polymer microcantilevers [44]. Through thermally pressing DFR onto pre-patterned Si wafers, suspended DFR membranes were created first. This is followed by UV exposure, post-drying, and development processes, which formed free-standing DFR microcantilevers (Figure 7a), effectively eliminating plastic deformation of the probes. By optimizing fabrication parameters, they successfully achieved a vertical deflection of only 1 μm at the free end of a 1000 μm long DFR microcantilever (Figure 7b), which can offer higher deflection sensitivity for biochemical sensing applications.

Compared to simple structured microcantilevers, specifically designed microcantilevers can achieve a greater range of functionalities [45,46,47]. Nilsen’s team [44] further realized the integration of a 3D polymer pillar and a piezoresistive metal layer (Nickel, Ni) on the surface of DFR microcantilevers (Figure 7c). Integrated structures like these hold broad application prospects in complex MEMS such as scanning tips, hollow polymer cantilevers, and piezoresistive read-out sensors.

#### 4.1.3. Waveguide Component Construction

Microwave frequency band is increasingly recognized as a valuable spectrum for future wireless systems. Meanwhile, as the operating frequencies keep increasing, the sizes of corresponding waveguide components are consistently shrinking. Traditional manufacturing methods for waveguide components, including Si micromachining [48,49], SU-8 lithography [50,51], and carbon nanotube (CNT) deposition [52], are either costly, complicated, time-consuming, or suffers from all the above-mentioned problems. Consequently, innovative manufacturing techniques for high-frequency devices have been continuously investigated.

**Figure 8:**
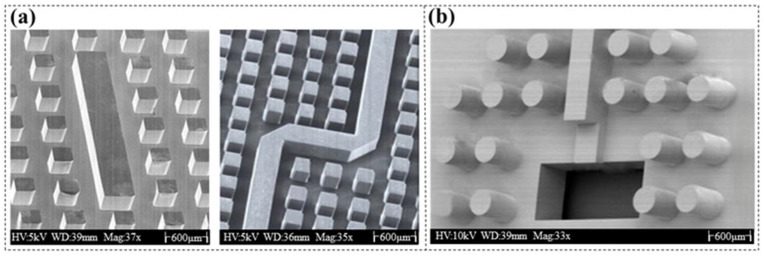
(**a**) SEM images of a straight gap ridge resonator (left) [53] and a bend ridge gap resonator (right) based on DFR [54]. (**b**) SEM image of a slot waveguide vibrating antenna with step structures, the DFR thickness ranges from 80 µm to 400 µm [55].

In the recent past, researchers [53,54] have explored the fabrication of ridge gap waveguide resonators operating above 200 GHz by using DFR (SUEX, 40 µm thick) (Figure 8a). Additionally, a gap waveguide slot array antenna [55] was created by laminating multilayer DFR (SUEX, 40 µm thick) and optimizing the exposure time of each layer, resulting in stepped structures with thickness ranging from 80 µm to 400 µm with “±10” μm tolerance (Figure 8b).

Table 2 presents performance comparisons between the DFR ridge gap resonator and those made from other materials in terms of unloaded quality (Q values) and loss, indicating that the DFR-based method delivered the best results [48,50,52,53]. The reasons are as follows: The main factor affecting the attenuation of gap waveguides is the conductivity of the walls, with the sidewall morphology of large aspect ratio components being a key structural element. The sidewall structure obtained through DFR exhibits higher precision in thickness uniformity and verticality, with a surface roughness of only 3.81 ± 0.5 nm. Consequently, this significantly enhances the performance of waveguide devices. So far, there are no further reports on the application of DFR in waveguide component manufacturing. Given its outstanding performance, it is anticipated that this will attract greater attention from researchers in this field [56].

### 4.2. MEMS Adhesive Encapsulation

DFR exhibits excellent low-temperature adhesion and is commonly used for MEMS adhesive encapsulation. Standard bonding techniques, including thermal bonding [57,58], adhesive bonding [59], and laser welding [60,61], typically require high bonding temperatures or additional adhesives.

**Figure 9:**
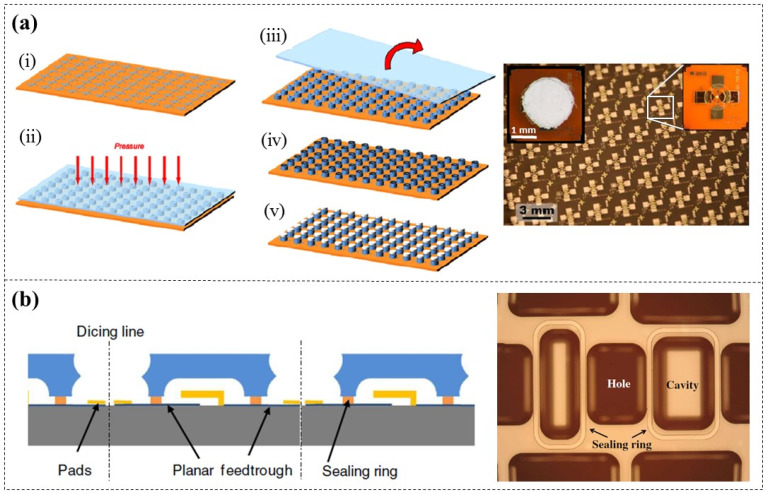
DFR-based adhesive encapsulation. (**a**) A chemical sensor [62]. The pre-patterned DFR rims were aligned with the polyimide foil containing the sensing devices and bonded together in a press for 3 min at 75 °C. Then, the PE handling layer on the DFR was removed and gas permeable membranes were deposited. This structure can protect the sensitive parts of the sensor from contamination or damage. (**b**) An RF MEMS device [63]. DFR rims were used to achieve packaging between the quartz caps and the RF MEMS substrate wafer. This structure can protect sensitive RF MEMS devices from moisture and mechanical damage during cutting and automatic processing.

Courbat et al. [62] discussed the application of DFR (PerMX3050, 50 µm thick) for encapsulating chemical sensors (Polyimide-based) to protect sensitive parts (Figure 9a), and Giacomozzi et al. [63] demonstrated the packaging and sealing of RF MEMS devices (Si-based) by using DFR (PerMX, 20 and 50 µm thick) and quartz caps (Figure 9b). Further studies have shown that DFR can achieve low-temperature bonding of various substrates, including metals, ceramics, and glass [64,65,66], facilitating the manufacturing of complex MEMS.

**Figure 10:**
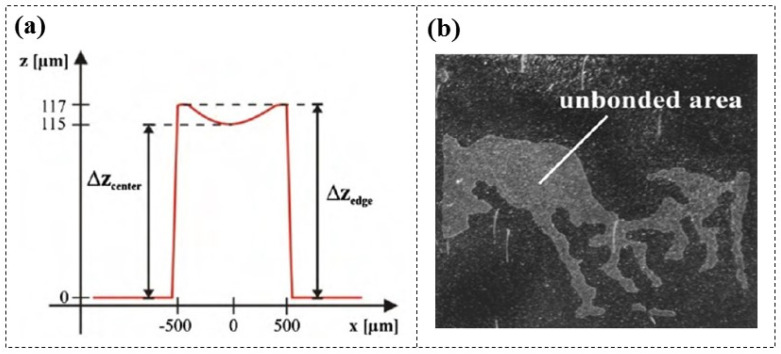
The impact of DFR line width on adhesive bonding performance [68]. (**a**) Surface profiler scan of a 1000 µm wide DFR line. (**b**) Optical micrograph of the poorly bonded chip.

DFR-based adhesive encapsulation can be completed in less than 4 h, with a sealing rate of 95%, and maintain good integrity over several months. However, researchers have found that when the line width of the patterned DFR (Ordyl SY330, 30 µm thick) exceeds 500 µm, a nearly constant superelevation (≈2 µm) appears at the edges of the structure [67,68] (Figure 10a). A possible reason for this is that the tensile layer stress leads to vertical contraction in the middle of the structure, and the resulting valleys often cause small air bubbles or unbonded areas at the bond interface (Figure 10b). There are no public reports on improving the sealing issues caused by uneven thickness of DFR lines at the centre and edges. However, drawing from similar challenges encountered in the application of SU-8 photoresist [69], it can be inferred that reducing the thickness of the DFR may enhance bonding performance.

**Figure 11:**
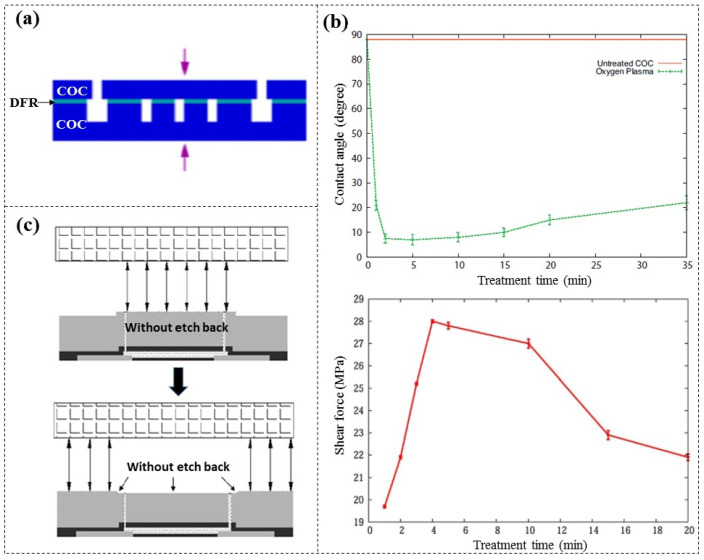
A COC chip based on DFR adhesive bonding [70]. (**a**) Schematic diagram and image of the COC chip. (**b**) Variation in contact angle on COC surfaces (top) and shear force test results of bonded chips (bottom) at a power rating of 25 W and an oxygen flow of 30 sccm. (**c**) Without etch-back planarization (top), lamination force directly compresses the nanochannels, causing water to be squeezed out. With etch-back planarization (bottom), the force is redirected, preventing water from being squeezed out of the nanochannels.

During the fabrication of a cyclic olefin copolymer (COC) chip (Figure 11a), El Fissi et al. [70] proposed treating the COC surfaces with oxygen plasma before bonding in order to improve their hydrophilicity and enhance the adhesion between DFR (Ordyl SY300, 30 µm thick) and cyclic olefin copolymer (COC) substrates. Surface contact angle observations and shear force tests indicated the optimal treating parameters, which include a power rating of 25 W, an oxygen flow of 30 sccm, and a treating time of 4 min (Figure 11b). This method is similar to the bonding technique employed for PDMS with various substrates, such as glass and Si [71,72,73].

When encapsulating the wet etched nanochannels, the traditional encapsulation processes usually require high-temperature conditions (>100 °C), which means that the nanochannels will be dried out, often resulting in stiction. Shen et al. [74] successfully encapsulated water-filled nanochannels using DFR (TMMF S2030, 30 µm thick). By employing etch-back planarization (Figure 11c), the water-filled region was made slightly lower (about 1 µm) than the surrounding area, effectively preventing water from being squeezed out during DFR lamination. With this method, stiction and bubble formation can be prevented as no drying steps were employed.

### 4.3. Flexible Electrode Insulation

With the development of flexible/wearable electronic technology, there is a growing demand for flexible transparent electrodes. The traditional choice has been indium tin oxide (ITO), but the brittleness and high processing cost limit its applications [75,76]. In recent years, silver nanowires (AgNWs) have been regarded as an ideal alternative to ITO due to their excellent mechanical flexibility, high transparency, and outstanding insulation [77,78,79].

**Figure 12:**
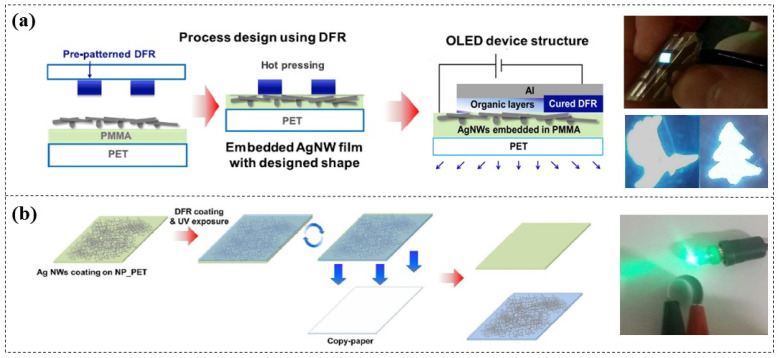
Applications of DFR-based insulation layers for the fabrication of (**a**) patterned AgNW electrodes [80,81] and (**b**) paper-based AgNW electrodes [82].

Kim et al. [80,81] explored the application of DFR (KL-1015, 15 µm thick) in manufacturing insulation layers for AgNW electrodes. Through a simple thermal pressing process, pre-patterned DFR were attached onto the AgNWs, resulting in desired patterned insulating electrodes for flexible OLED lighting (Figure 12a). Additionally, they further developed paper-based AgNW electrodes using DFR (Figure 12b) and demonstrated that the conductivity of these electrodes remains nearly constant after 1000 bending cycles, showing excellent mechanical stability and flexibility [82]. Similarly, Weltin et al. [83] adopted this approach to create flexible electrodes for a microsensor.

The DFR can serve not only as an insulating layer for the fabrication of flexible electrodes but also as a mask for etching electrodes, which will be discussed more later in this paper.

## 5. Applications of DFR as Mould Processing Materials

### 5.1. Metal Electroplating Moulds

**Figure 13:**
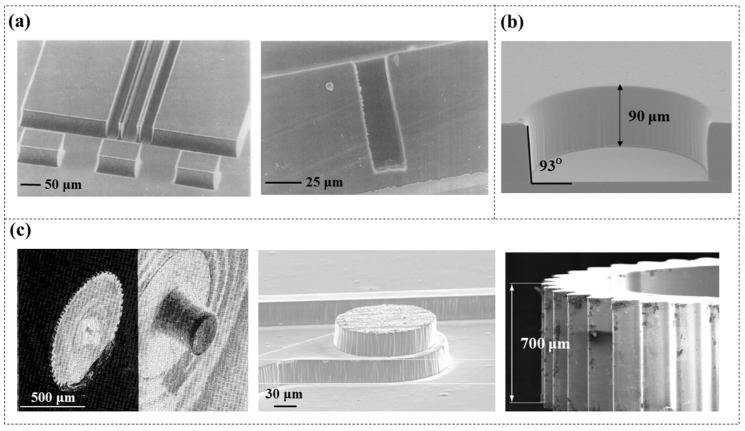
(**a**) DFR-based electroplating moulds fabricated by RIE (left) and laser ablation (right) techniques [87]. (**b**) A DFR-based electroplating mould (90 µm deep) fabricated by UV lithography [3]. (**c**) Electroplated Ni parts created via multilayer DFR moulds [3,88,89].

The LIGA (Lithografie Galvanoformung Abformung) process has been shown to be capable of producing metal electroforming moulds with high aspect ratio, but requires special masks and high-intensity synchrotron X-ray radiation source [84,85,86]. Zhu et al. [87] first employed DFR (Riston, 30 µm thick) to fabricate electroplating moulds through reactive ion etching (RIE) and excimer laser ablation techniques (Figure 13a). However, both of these processing procedures remain relatively complex.

Researchers have further developed what is called “poor man’s LIGAs”, which creates electroplating moulds based on DFR and UV lithography, enabling low-cost and rapid large-scale patterning. Figure 13b shows a 90 µm deep DFR (Ordyl, 20 and 100 µm thick) mould of an inertial micromachined disc, which shows uniform height and nearly vertical sidewalls [3]. Later, more studies reported the feasibility of achieving high aspect ratio electroplated structures using DFR moulds [88,89]. However, it was found that the surfaces of these multilayer electroplated structures often exhibit irregularities (wave) or photoresist residue, leading to an increase in roughness, as shown in Figure 13c. The irregularities on the top may be due to the difficulty in stopping the plating process at the right time, while those on the sidewalls mainly resulted from residual DFR polymers. Therefore, further grinding and polishing steps are necessary for removal.

### 5.2. Microfluidic Moulds

The utilization of moulds for the fabrication of microfluidic chips is a commonly employed method in chip processing. These microfluidic moulds are commonly fabricated through deep reactive ion etching (DRIE) [90] or SU-8 lithography on Si/glass substrates [91,92], but both methods are relatively time-consuming.

**Figure 14:**
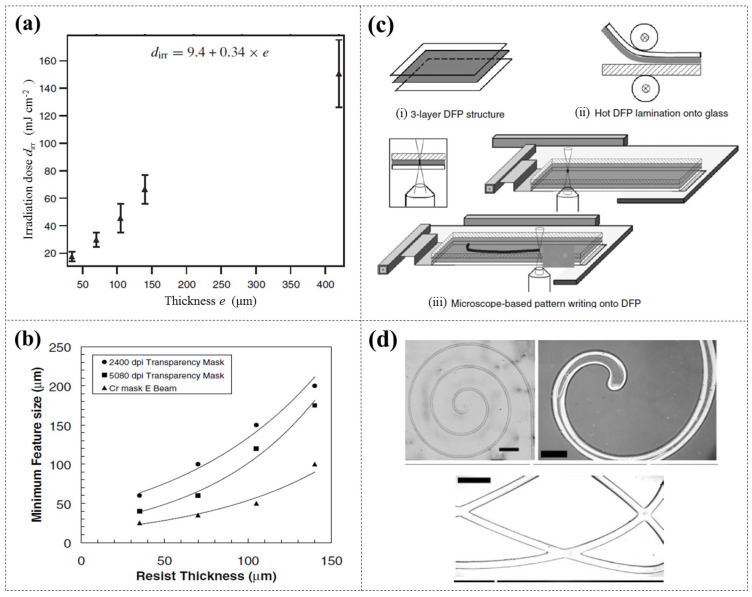
(**a**) The optimal UV exposure dose at varying DFR thicknesses [93]. (**b**) The relationship curve between the minimum feature size and DFR thickness when using different masks [94]. (**c**) Microscope-based pattern writing onto DFR, and (**d**) example patterns produced [95].

Stephan et al. [93] reported the use of DFR (Etertec HQ-6100, 35 μm thick) and UV lithography to fabricate microfluidic moulds, which were employed to replicate PDMS chips. They observed that the optimal exposure dose is primarily linearly related to the DFR thickness (Figure 14a), slightly with the type of substrate used. Leech et al. [94] determined the resolution limits of DFR (Shipley 5038, 35 μm thick) as a function of three different types and resolutions of masks, achieving a minimum feature size of 20 μm (Figure 14b). Leigh et al. [95] developed a maskless DFR (Ordyl SY 330, 30 μm thick) patterning system that employed a fluorescence microscope equipped with a programmable X-Y stage, enabling the creation of arbitrary patterns ranging from 40 μm to 700 μm (Figure 14c,d).

**Figure 15:**
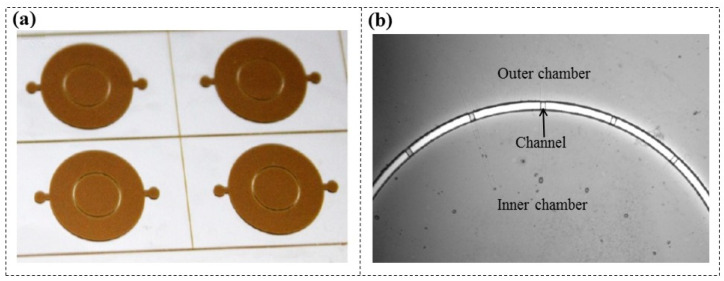
(**a**) Multilayer DFR microfluidic moulds after adding the post-exposure baking step, and (**b**) the microscope image [99].

When using multilayer DFR with different patterns to create complex microfluidic moulds, laminating onto pre-patterned DFR can be challenging, potentially damaging pattern features and causing poor adhesion between layers [96,97,98]. Koucherian et al. [99] proposed a method involving post-exposure baking to address this challenge. Each layer of DFR (ADEX, SUEX) required baking after exposure to generate visible images suitable for subsequent layer alignment, without the need for any developing agents. After completing all lamination processes, development was performed. This approach effectively reduced the damage to DFR patterns from multilayer lamination, especially for fine patterns with small areas of photoresist. They fabricated a neuronal chemotaxis device consisting of two chamber layers and a narrow channel (50 μm wide) layer, as illustrated in Figure 15, demonstrating excellent pattern fidelity and alignment accuracy.

**Figure 16:**
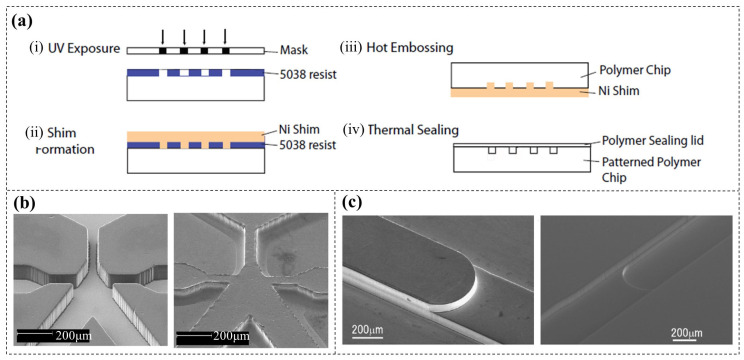
(**a**) Schematic diagram of rigid microfluidic chip processing based on DFR moulds, and (**b**) SEM images of a DFR mould (left) and the replicated Ni shim (right) used for droplet generation [94]. (**c**) SEM images of a DFR mould (left) and the replicated Ni shim (right) used for microflow injection analysis (µFIA) [100].

Although DFR moulds are highly convenient for the soft replication of PDMS chips, they are not suitable for the hot pressing of rigid chips (e.g., glass, PMMA). Leech et al. [94] first reported the application of DFR (Shipley 5038, 35 μm thick) moulds in the fabrication of rigid droplet-generating chips. A DFR mould with microstructures was fabricated by UV lithography and replicated as a Ni shim with higher hardness. Then, the patterns were then transferred from Ni shim onto Plexiglas (PMMA) by hot embossing, and finally the chip was encapsulated (Figure 16a). The surfaces of both the DFR mould and the replicated Ni shim were notably smooth, achieving features with a resolution of 50 μm and an aspect ratio of 1.4 (Figure 16b). Similarly, Ito et al. [100] employed this method (DFR: WBR 2075, 75 μm thick) to fabricate PMMA chips for microflow injection analysis (µFIA) (Figure 16c).

## 6. Applications of DFR as Sacrificial Mask Materials

The concept of sacrificial layers was first proposed by researchers at Westinghouse Electric Company in 1967 for fabricating metal cantilever beam structures [101]. This technology has since found widespread application in the field of micromachining [102,103,104]. This section will discuss the applications of DFR as sacrificial mask materials in micromachining.

### 6.1. Sacrificial Masks for Chemical Etching

**Figure 17:**
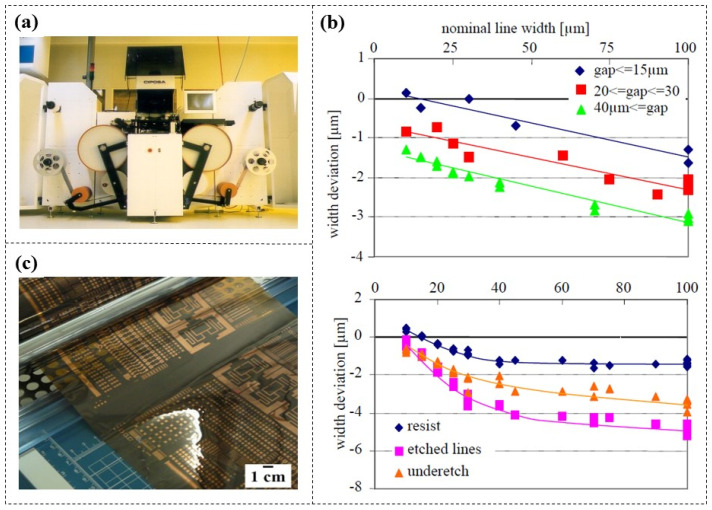
(**a**) A roll-to-roll flexible lithography system using DFR masks, and (**b**) the width deviation of DFR lines (top) and etched copper lines (bottom) from nominal values [109]. (**c**) Image of a flexible printed copper foil circuit [111].

Flexible printed circuits (FPCs) emerged in the 1960s, known for their flexibility, allowing them to be folded or rolled for compact integration into devices, and significantly reducing system size [105,106,107]. Common methods for FPC processing, including inkjet printing, screen printing, and gravure printing, are not well-suited for industrial-scale production [108]. Drost et al. [109] employed DFR (MX5015, 15 μm thick) to fabricate chemical etching mask layers and developed a roll-to-roll flexible lithography system (Figure 17a). Copper-coated foils were used as the substrates and DFR was laminated on the copper surfaces using rubber rollers at 85 °C. Following UV exposure, the developing solution was sprayed by nozzles onto the foil with a pressure of 1.4 bar. Finally, after rinsing with deionized water, they successfully achieved lithography on copper foil lengths of up to 30 m. Furthermore, they proved that the width deviation of the DFR lines and etched copper lines from the nominal values was dependent on distance between the lines; underetching also followed this trend (Figure 17b). Therefore, it is essential to consider this factor in circuit component layout rules to achieve accurate dimensional compensation. This method has also been employed in other studies [110,111] for the printing and integration of FPC (Figure 17c).

In addition to the previously mentioned use of DFR as insulating layers for fabricating flexible electrodes, it can also serve as sacrificial masks for flexible electronics [12,112].

**Figure 18:**
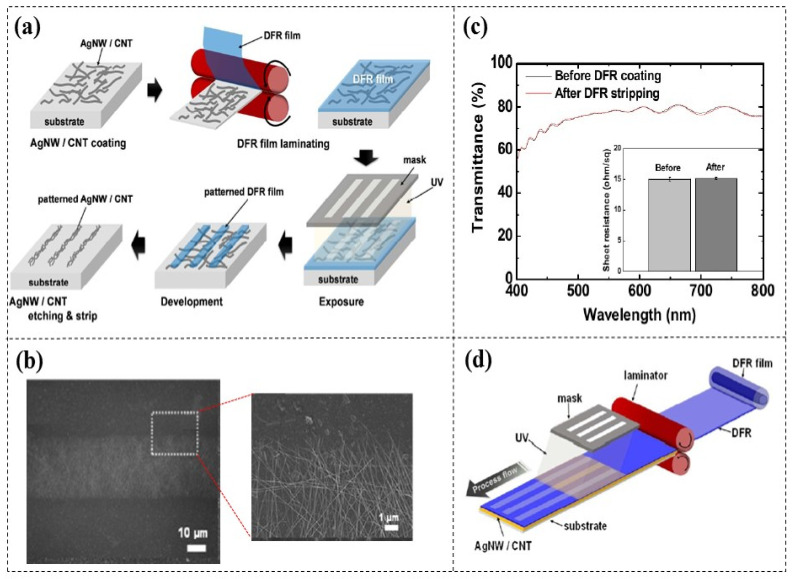
Chemical etching of AgNW electrodes based on DFR masks [113]. (**a**) Schematic diagram of the etching process. (**b**) SEM image of a patterned AgNW electrode with a width of 30 µm. (**c**) Optical transmittance test results for AgNW networks before and after DFR patterning. (**d**) Conceptual diagram of a roll-to-roll processing system for AgNW electrodes based on DFR masks.

Amicucci et al. [79] and An et al. [113] demonstrated the chemical etching of flexible AgNW electrodes using DFR (KL-1015, 15 µm thick) masks. (Figure 18a). When the pattern length is less than 1 cm, the minimum feature size of 30 µm can be achieved, which meets the typical requirement for flexible electrode patterns (around 100 µm) and exhibits clear edges (Figure 18b). Furthermore, the investigation revealed that the patterning process of DFR did not significantly affect the conductivity and optical transmittance of AgNWs (Figure 18c). This technology shows great potential for high throughput roll-to-roll manufacturing of flexible electrodes (Figure 18d). Bhattacharya et al. [114] successfully achieved large-area selective etching of ITO films using DFR (PM 240, 38 μm thick), and further improved the pattern resolution by reducing DFR thickness and employing a spray development method.

**Figure 19:**
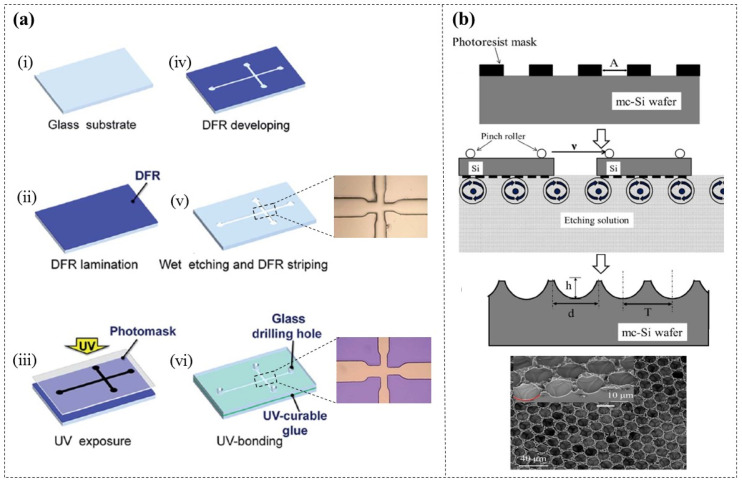
Schematic diagrams of DFR masks for (**a**) etching microchannels on glass substrates [119], and (**b**) creating honeycomb-like textures on mc-Si solar cells [121].

The masks used for chemical etching of rigid substrates are typically created by depositing mask layers (e.g., chromium, toner.) [115] or through spin-coating liquid photoresist [116,117,118], both of which can be costly and time-consuming. In 2015, DFR (Etertec HT-115T, 40 µm thick) masks were first reported to be used for the chemical etching of microchannels on glass substrates [119] (Figure 19a). The observation results showed clear and orderly edges of the glass microchannels, with no resist residues. However, there was a certain deviation between the dimensions in the mask design and those of the actual glass channels. For instance, a channel with a minimum width of 50 µm in the mask corresponded to a width of 85 µm on the glass substrate. Therefore, further improvements in processing accuracy are needed. Zhang H. et al. [120] employed this method to etch honeycomb-like textures on multicrystalline silicon (mc-Si) solar cells, aiming to reduce the reflectance of the cell surface and prolong the lifetime of charge carriers. Later, they further developed a true single-face texturing method for mc-Si solar cells [121] (Figure 19b), eliminating the backside protection and cleaning steps. This technology has led to an average efficiency increase of 0.34% in mc-Si solar cells, which is significant for enhancing their performance.

Additionally, Kikuchi et al. [122] reported the use of DFR (Nafion 117, 183 µm thick) masks for electroless plating to create patterned electrodes on ionic polymer–metal composites (IPMCs). However, there were also certain deviations between the width of the electrodes formed and those of the mask. The primary reason was attributed to the diffusion of metal complexes beneath the DFR pattern during adsorption. Reducing the thickness of the DFR can help minimize errors in electrode line width.

### 6.2. Sacrificial Masks for Electrochemical Micromachining (EMM)

EMM is a process that removes material from the anode workpiece through selective electrochemical reactions in an electrolytic cell. Compared with traditional chemical etching, EMM enables the machining of complex shapes on objects regardless of their hardness and surface roughness [123,124,125]. Mask-based EMM is a commonly employed method; however, mask processing faces the same challenges as that encountered in conventional chemical etching [34,115,126,127,128].

**Figure 20:**
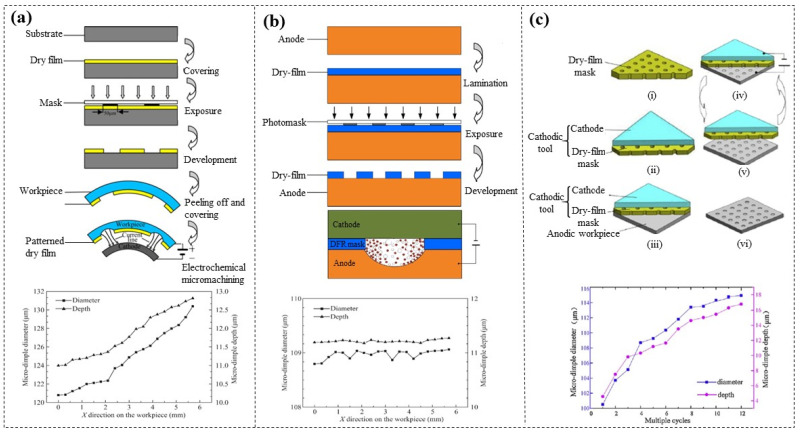
(**a**) EMM of microdimple arrays on cylindrical inner surfaces based on DFR masks [129]. (**b**) SLEMM of microdimples [130]. (**c**) Movable DFR mask EMM of microdimples [131].

Qu et al. [129] coated patterned DFR (GPM 200, 50 µm thick) on cylindrical inner surfaces to serve as EMM masks, successfully etching microdimple arrays in just 9 s. However, the electric field strength at the edges was higher than that in other regions, resulting in poor uniformity of the microdimple arrays (Figure 20a). A new method, sandwich-like electrochemical machining (SLEMM), has been proposed [130]. By firmly attaching the DFR (GPM 200, 50 µm thick) mask to the anode workpiece, the electrolyte on the workpiece surface is confined within a defined area by the DFR mask, effectively preventing overcutting of the microdimple and ensuring dimensional uniformity (Figure 20b). In addition, this team also proposed a novel EMM system involving a movable cathode tool, which consists of a metal cathode and attached patterned DFR masks [131] (Figure 20c). The cathode tool moved downward to make close contact with the anode; after etching is complete, it returned upward. The unique advantage of this method lies in the fact that the DFR masks can be reused repeatedly, and the results from multi-cycle EMM experiments demonstrated good machining accuracy.

## 7. Conclusions and Discussion

Compared to commonly used SU-8 photoresist, DFR exhibits significant advantages in many aspects, as summarized in Table 3. These unique benefits have been harnessed in numerous applications in micromachining.

Although significant progress has been achieved, DFR-based micromachining technology still faces some challenges. In this review, we discuss the current progress, key challenges, and future prospects of DFR in micromachining applications, as shown in Table 4. It is expected that this review can provide guidance for the future applications of DFR in more fields and serve as a reference for more in-depth research on DFR.

## Figures and Tables

**Table 1:** Summary of comparisons between positive DFR and negative DFR.

Features	Positive DFR	Negative DFR
Lithography pattern	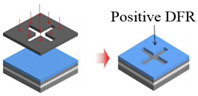	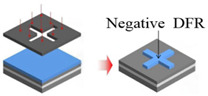
Thermal stability	Easy to soften at high temperature, storage at 2~8 °C.	High thermal resistance, suitable for hot pressing, storage at 5~20 °C.
Adhesion	Common adhesion performance, suitable for smooth surfaces.	Good adhesion performance, also applicable for rough surfaces.
Exposure principle	Photodecomposition reaction.	Cross-linking reaction.
Developer solution	0.1~0.4% (*w*/*w*) C_4_H_13_NO/KOH solution.	1~2% (*w*/*w*) Na_2_CO_3_ solution/C_6_H_12_O_3_/C_8_H_10_
Resolution	Achievable at the nanoscale	Typically >=10 μm, due to swelling deformation during development.
Corrosion resistance	Poor	Good
Cost	High	Low
Application fields	Ultrafine structure micromachining, such as logic chips and memory devices, etc.	Standard structure micromachining, such as packaging, MEMS devices, and PCB manufacturing, etc.

**Table 2:** Performance comparisons of DFR ridge gap resonators with those made from other materials.

Materials	First Resonance (GHz)	Unloaded Quality (Q)	Loss (dB/m)	Ref.
	Second Resonance (GHz)			
Si	234	642	0.033	[48]
	284	628	0.043	
SU-8	234	319	0.067	[50]
	284	628	0.041	
CNT	234	274	0.079	[52]
	284	518	0.051	
DFR	234.6	656	0.032	[53]
	284	786	0.033	

**Table 3:** Summary of comparisons between SU-8 and DFR.

Photoresist	SU-8	DFR
Clean environment and with expensive equipment	Necessary	Unnecessary
Substrate flatness	Only suitable for coating on flat surfaces	Suitable for laminating on both curved and uneven surfaces
Lamination temperature	>=95 °C	50~85 °C (the required temperature gradually decreases from the first layer)
Processing time	Coating and baking are necessary and these processes will take about 30 min	Coating and baking are not needed, and the lamination will take about 3~5 min
Uniformity of thickness	Non-uniform thickness	Uniform thickness
Pattern resolution	<=1 μm	>=10 μm
Aspect ratio	Up to 10	<=7 (Free-standing structure) <=5 (Channel structure)
Large area processing	Not suitable due to significant edge effects	Suitable
Solvent waste	More than 95% of the photoresist solvent is wasted	No waste

**Table 4:** Summary of the progress and prospects of DFR in micromachining.

Current Progress	Key Technological Challenges	Future Opportunities
Achieved extensive applications in micromachining, including microstructure definition, MEMS packaging, mould processing, and microelectrode fabrication, etc. Provided and validated many key processing parameters for different types of DFR. Easily achieved high aspect ratio microstructures through multilayer lamination technology. Explored various methods to enhance the adhesion of DFR, bonding strength, and pattern accuracy, including low-temperature lamination, vacuum plasma cleaning, and post-exposure baking, etc. Significant improvements have been achieved through these approaches.	Consistent conclusions on the key processing parameters have not yet been reached for different types of DFR. For wide-line structures, lamination often results in uneven thickness, which seriously affects the performance of microdevices. Lamination of patterned DFR may cause sagging in the upper layer and obstruct the channels of the lower layer. Precise alignment for multilayer patterns remains difficult. To achieve a greater aspect ratio, more layers need to be laminated, and the inconsistent shrinkage between layers will significantly impact the structural integrity.	Further explore methods to improve the precision of microfabrication, such as reducing the deformation of multilayer DFR, enhancing the adhesion, and improving the alignment accuracy. Striving to achieve higher aspect ratios to meet a broader range of microfabrication requirements. There is limited research on the reusability and service life of DFR to date when used as non-sacrificial materials (structural materials and mould processing materials). Given its significance for future industrial applications, this aspect needs more attention.

## Data Availability

No new data were created or analyzed in this study.
